# Alkali-Equivalent-Dependent Dual Role of Sodium Chloride in Alkali-Activated Slag Cement: From Synergistic Activator to Competitive Inhibitor

**DOI:** 10.3390/ma19061166

**Published:** 2026-03-17

**Authors:** Nan Ding, Zhenyun Cheng, Hua Lei, Bo Fu

**Affiliations:** 1 College of Civil Engineering, North Minzu University, Yinchuan 720021, China; 20247585@stu.nmu.edu.cn (N.D.); 20237539@stu.nmu.edu.cn (H.L.); 2 College of Civil Engineering, Hefei University of Technology, Hefei 230009, China; 2016035@nmu.edu.cn

**Keywords:** alkali-activated slag cement, alkali equivalent, chloride binding, Friedel’s salt, C-(A)-S-H gel, ionic strength, hydration mechanism

## Abstract

The cement industry is a major contributor to global CO_2_ emissions, necessitating the development of low-carbon alternatives, such as alkali-activated slag cement (AAS). This study investigates the feasibility of using NaCl and NaOH as co-activators for granulated blast furnace slag (GBFS), focusing on the alkali-equivalent-dependent role of NaCl. At a low-alkali equivalent (2% Na_2_O), incorporation of ≤4 wt% NaCl enhanced ionic strength, promoted slag dissolution, and accelerated C-(A)-S-H gel formation, increasing 28-day compressive strength by up to 21%. In contrast, at a high-alkali equivalent (4% Na_2_O), NaCl addition induced competitive binding of Cl^−^ with aluminate species, inhibiting C-(A)-S-H formation and reducing strength by up to 18% at 10 wt% NaCl. The optimal NaCl dosage for strength improvement was 1–4 wt% under low alkalinity and 1–2 wt% under high alkalinity. Microstructural analyses (XRD, FTIR, TG-DTG, SEM-EDS) confirmed that NaCl promotes Friedel’s salt formation under both conditions, but its effect on the primary gel phase is alkalinity dependent. This work provides a theoretical basis for utilizing industrial NaCl by-products in low-carbon cement design and highlights the importance of alkalinity control in achieving synergistic activation.

## 1. Introduction

The cement industry accounts for approximately 7–8% of global CO_2_ emissions from fossil fuel combustion and related processes [1,2]. Considering fuel and transportation, about 0.83 tons of CO_2_ are emitted per ton of cement produced [2]. Therefore, developing environmentally friendly alternatives to Portland cement (PC) is essential for reducing environmental pressure. Alkali-activated slag cement (AAS) is a promising green binder that uses industrial by-product slag as the main raw material and avoids high-temperature clinker calcination, significantly reducing energy consumption and CO_2_ emissions [3].

The performance of AAS depends largely on the activator. Common activators like sodium hydroxide and water glass are effective, but they are energy-intensive, corrosive, and generate high CO_2_ emissions [4,5]. This has led to growing interest in novel activators with lower alkalinity. Recent studies have explored various alternatives. Wan et al. [6] developed a mixed activation method using Na_2_CO_3_ to partially replace NaOH, improving interfacial structure and mechanical properties. Wang et al. [7,8] used Na_2_SO_4_ as an activator for steel slag–GBFS blends, showing that sulfate promotes C-S-H formation and optimizes pore structure. Kamali et al. [9] investigated high-concentration brine combined with low-concentration NaOH to activate GBFS, achieving a 28-day compressive strength of 28.0 MPa. Table 1 summarizes recent studies on alternative activators in alkali-activated systems, providing an overview of the current research landscape.

From a thermodynamic perspective, chloride behavior in alkali-activated systems is governed by dissolution–precipitation equilibria in the Ca-Al-Cl system. The formation and stability of Friedel’s salt (3CaO·Al_2_O_3_·CaCl_2_·10H_2_O) play a critical role in chloride immobilization [25]. Friedel’s salt precipitation is controlled by the activity ratio of Cl^−^ to OH^−^ in pore solution and the activity of Al(OH)_4_^−^ [26]. Calcium- and aluminum-rich components in AAS enhance chloride binding by increasing the availability of Ca^2+^ and dissolved Al species, promoting the formation of Friedel’s salt and hydrotalcite-like LDH phases [27]. Regarding slag dissolution kinetics, ionic strength and surface complexation are key factors determining slag reactivity [28].

Notably, large amounts of saline wastewater (mainly NaCl) are generated by various industries, posing severe disposal challenges [29]. If such wastewater could be used as an activator component in AAS, it would achieve the dual goal of waste treatment by waste. Previous studies have shown that Na^+^ and Cl^−^ can promote calcium chloroaluminate phase formation, strengthening the matrix and improving early strength [30]. Chloride incorporation also benefits durability by promoting C-(N)-A-S-H gel densification and inducing hydrotalcite formation, which binds Cl^−^ and delays harmful ion ingress [31]. Although NaCl introduces chloride ions, they can be effectively fixed as stable chloroaluminate phases without significantly increasing free chloride concentration [32]. However, existing studies have mainly focused on chloride immobilization and durability under single alkalinity conditions. Systematic understanding of how Cl^−^ regulates slag dissolution and gel structure evolution under varying alkalinity remains limited. Under low alkalinity, Cl^−^ may exhibit synergistic activation by increasing ionic strength and promoting slag dissolution. Under high alkalinity, Cl^−^ may compete with OH^-^ for reaction sites and preferentially bind with Al^3+^, affecting C-(A)-S-H gel formation and stability.

To further advance the understanding of the coupled effects of alkalinity and chloride ions on AAS hydration, the present study establishes a dual-variable system involving alkalinity (2% and 4% Na_2_O) and NaCl dosage (0–10%). The coupled effects on macroscopic properties and microstructure of AAS are systematically investigated through fluidity, setting time, compressive strength tests, and multiple characterization techniques, including XRD, FTIR, TG-DTG, and SEM-EDS. The alkalinity-dependent dual role of Cl^−^ and its regulatory mechanism on C-(A)-S-H gel evolution are elucidated. This study provides a unified interpretative framework for understanding AAS behavior in chloride-containing environments and proposes a feasible strategy of partially replacing NaOH with NaCl under low-alkalinity conditions, offering theoretical support for low-carbon activator design and industrial by-product salt utilization.

## 2. Experiment

### 2.1. Raw Materials

**Figure 1:**
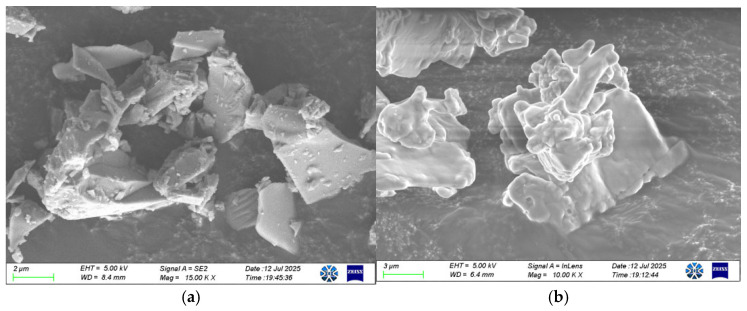
SEM micrographs of raw materials. (**a**) GBFS; (**b**) NaCl.

**Figure 2:**
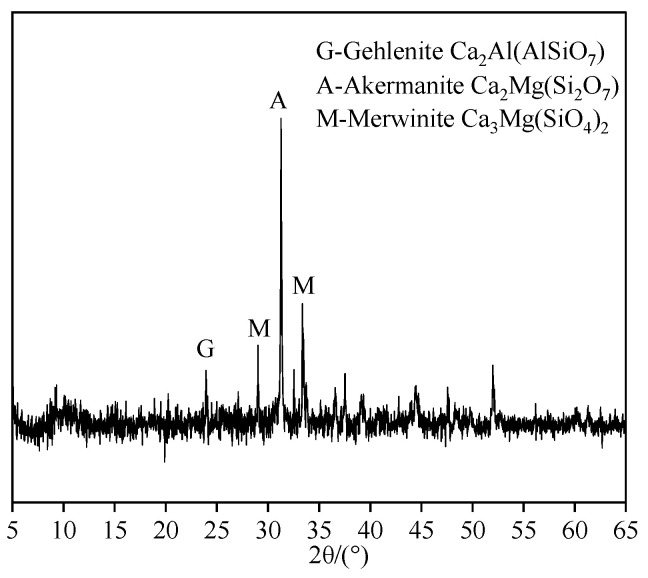
X-ray diffraction pattern of GBFS.

Granulated blast furnace slag (GBFS), a gray-white powder, was sourced from Ningxia Qingtongxia Cement Co., Ltd. (Qingtongxia, China). Sodium hydroxide (analytical grade) was used as the primary activator, sourced from Ningxia Tianshengyuan Biotechnology Co., Ltd. (Shizuishan, China). Sodium chloride (analytical grade) was purchased from Tianjin Dingshengxin Chemical Co., Ltd. (Tianjin, China). The microstructure of the raw materials is shown in Figure 1. The GBFS particles exhibit an amorphous morphology, while NaCl consists of irregularly stacked crystalline particles. The XRD pattern of GBFS is shown in Figure 2. The broad hump centered between 25° and 35° (2θ) is characteristic of a dominant amorphous phase, confirming the high potential reactivity of GBFS.

**Figure 3:**
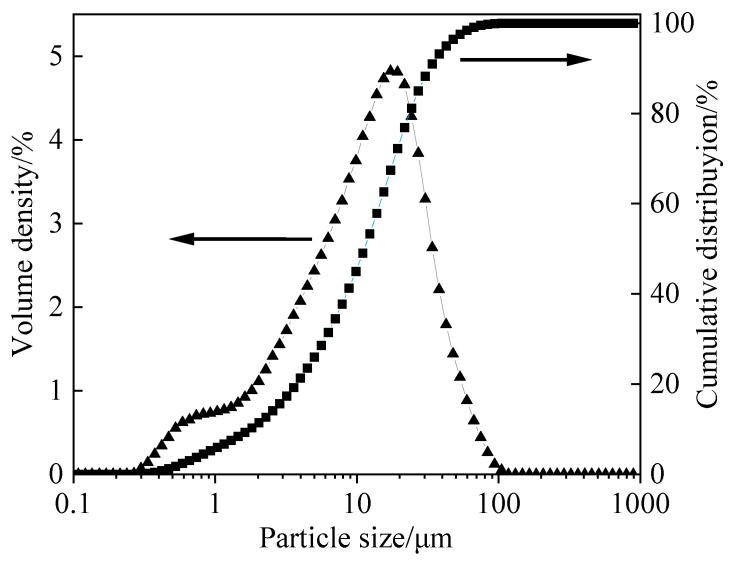
Particle size distribution of GBFS.

The chemical composition of the raw materials was determined by X-ray fluorescence (XRF, Zetium, Malvern Panalytical, Malvern, UK). As shown in Table 2, the main oxides in GBFS are SiO_2_, CaO, Al_2_O_3_, and MgO, which together constitute 94.64 wt% of its total mass. The particle size distribution of GBFS was measured using a Smart Laser Particle Size Analyzer (Bettersize 2000, Bettersize Instruments Ltd., Changsha, China). As shown in Figure 3, the particle size of GBFS exhibits a continuous distribution, with the median particle size (D50) of GBFS being 12.64 µm and a specific surface area of 450 m^2^/kg.

### 2.2. Sample Preparation

#### 2.2.1. Mix Proportions

The mix proportions of AAS are shown in Table 3. GBFS was used as the solid precursor, while sodium hydroxide and sodium chloride served as activators. The water-to-binder (w/b) ratio was fixed at 0.38 for all mixtures. Sodium hydroxide pellets and deionized water were used to prepare alkaline solutions with alkali equivalents of 2% and 4% Na_2_O (by mass of GBFS); the corresponding NaOH molarities were approximately 1.3 mol/L and 2.6 mol/L, respectively (calculated based on the solution volume). Before paste preparation, the pre-weighed sodium hydroxide was first added to deionized water and continuously stirred until completely dissolved. The resulting NaOH solution was immediately sealed with plastic wrap to prevent carbonation and then cooled to room temperature (20 °C). Subsequently, NaCl was added at 0%, 1%, 2%, 4%, 6%, 8%, and 10% by mass of GBFS. The pre-weighed sodium chloride was dissolved in the cooled NaOH solution under magnetic agitation until fully dissolved, forming a homogeneous NaCl-NaOH composite activator. The total Na^+^ and Cl^−^ concentrations for each mix are provided in Table 3.

#### 2.2.2. Preparation of Mortar Samples

Mortar samples were prepared according to the mix proportions in Table 3, with a binder-to-sand mass ratio of 1:2. Standard sand (ISO 679 [33]) was used. Mixing was performed in a planetary mixer according to the Chinese national standard GB/T 17671-2021 [34], with the sole modification that the mixing water was replaced by the NaCl-NaOH composite activator solution. Fresh mortar was cast into 40 mm × 40 mm × 40 mm molds, compacted on a vibration table, and covered with plastic film. After 24 h of curing in a standard curing chamber (temperature 20 ± 2 °C, relative humidity ≥ 95%), specimens were demolded and returned to the curing chamber until testing. For each mix and age, three specimens were tested, and the average compressive strength with standard deviation was reported.

#### 2.2.3. Preparation of Paste Samples

The AAS paste was prepared according to the mix ratios shown in Table 3. First, the pre-weighed granulated blast furnace slag (GBFS) was placed into the mixing bowl of a planetary mixer. The mixer was started, and dry mixing was carried out at low speed (140 ± 5 r/min, planetary rotation) for 30 s. Then, while maintaining low-speed mixing, the NaCl–NaOH composite solution was uniformly added into the mixing bowl within 15 s. Immediately after the addition of the solution, the mixing speed was increased to high speed (285 ± 5 r/min) and continued for 60 s until a homogeneous paste was obtained. After mixing, part of the fresh paste was used for fluidity and setting time tests, while the remaining portion was cast into cylindrical plastic tubes with a diameter of 8 mm. The tubes were then placed in a standard curing chamber (relative humidity ≥ 95%, temperature 20 ± 1 °C) and cured until the predetermined age. At the specified curing age, the specimens were removed, crushed, and immersed in isopropanol to terminate hydration. The isopropanol was replaced with fresh solution after 1 h and again after 24 h. After 3 days of immersion, the samples were taken out and vacuum-dried at 40 °C to a constant mass. Two to three relatively flat fragments were selected for SEM-EDS analysis. The remaining samples were ground and passed through a 75 μm square-hole sieve to obtain powder samples for XRD, FTIR, and TG-DTG analyses. To ensure the reproducibility of the experimental results, all sample preparations were conducted under controlled environmental conditions (temperature 25 ± 1 °C, relative humidity 50 ± 5%). For paste samples, an additional 30 s of manual stirring was performed before casting into molds to enhance homogeneity.

### 2.3. Testing Methods

The flowability of the fresh paste was tested according to the Chinese national standard GB/T 8077-2012 [35]. The prepared paste was quickly poured into a truncated cone mold, and the surface was leveled with a trowel. The mold was then lifted vertically, allowing the paste to flow freely on a smooth glass plate. After 30 s, the maximum diameter in two perpendicular directions was measured, and the average value was calculated as the flowability. For each mixture, the test was performed in three independent repetitions, and the results are expressed as mean ± standard deviation. The setting time was determined using a Vicat apparatus in accordance with GB/T 1346-2011 [36]. Two Vicat apparatuses were used in parallel; the initial and final setting times were averaged, and if the difference between the two measurements exceeded 15 min, the test was repeated. Compressive strength was tested on mortar samples. Prior to testing, specimens were removed from the standard curing chamber, and the surfaces were wiped dry. An axial load was applied using a press machine at a loading rate of 2400 ± 200 N/s until failure. The maximum failure load was recorded, and compressive strength was calculated. For each mixture and testing age (1, 3, 7, and 28 days), six specimens were prepared. After excluding outliers, the average of no fewer than four valid data points was taken as the final result, and all results are expressed as mean ± standard deviation.

XRD testing was conducted using a Smartlab SE X-ray diffractometer manufactured by Rigaku Corporation (Tokyo, Japan). The testing parameters were set with a tube voltage of 40 kV, a tube current of 200 mA, Cu target radiation, a scan speed of 1°/min, and a diffraction angle 2θ scan range of 5–65°. FTIR testing was conducted using a Nicolet iS50 Fourier Transform Infrared Spectrometer manufactured by Thermo Fisher Scientific (Waltham, MA, USA). The samples were prepared using the potassium bromide (KBr) pellet method, where 1 mg of the sample powder was weighed and uniformly mixed with 100 mg of KBr. The testing scan range was 4000–400 cm^−1^, with a spectral resolution of ≤0.09 cm^−1^, and 32 scans were accumulated. TG-DTG testing was conducted using an STA 449 F3 simultaneous thermal analyzer manufactured by Netzsch Instrument GmbH (Selb, Germany). The temperature range for the thermal analysis was from 30 °C to 1000 °C, with a heating rate of 10 °C/min. The purging gas used was argon (flow rate 50 mL/min), and the protective gas was nitrogen (flow rate 20 mL/min). SEM-EDS testing was conducted using a SIGMA 500 field emission scanning electron microscope integrated system manufactured by Carl Zeiss (Oberkochen, Germany).

## 3. Results and Discussion

### 3.1. Flowability and Setting Time

#### 3.1.1. Fluidity

**Figure 4:**
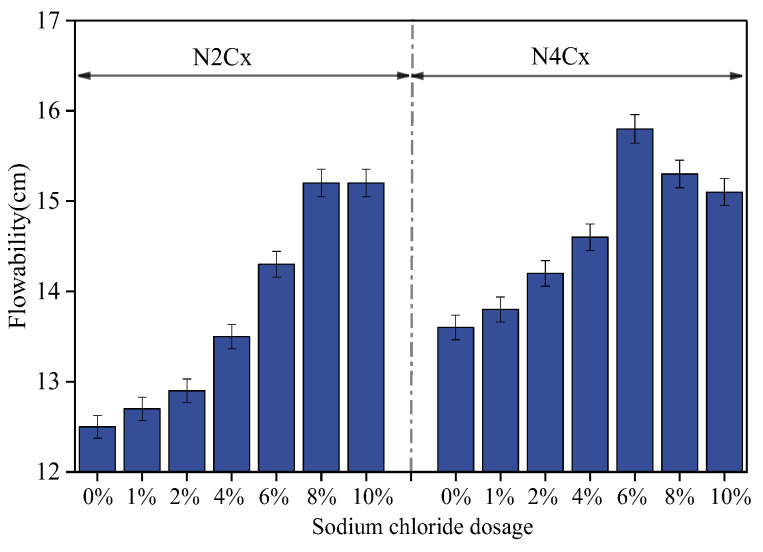
Effects of alkali equivalent (Na_2_O%) and NaCl dosage on the fluidity of AAS.

Figure 4 shows the variation in flowability of the NaCl-NaOH–slag composite paste with different alkalinity equivalents as a function of NaCl dosage. As shown, at low alkalinity (2%), the flowability of the paste increases with the increasing NaCl dosage. This may be due to the fact that under low-alkalinity conditions, the additional Cl^−^ ions introduced improved the uniformity of the surface charge distribution on the slag particles, enhanced the repulsive forces of the double electric layer structure on the slag surface, and increased the dispersion of the slag particles in the paste [37], resulting in increased flowability.

In contrast to the low-alkali equivalent, under a high-alkali equivalent (4%) condition, the flowability of the system first increases and then decreases with increasing NaCl dosage. When an appropriate amount of NaCl is added (≤4 wt%), the introduction of Na^+^ and Cl^−^ alters the surface charge distribution of the slag particles. Under highly alkaline conditions, a large amount of Na^+^ has already been adsorbed onto the slag surface. With the incorporation of a moderate amount of NaCl, Cl^−^ may undergo specific adsorption, thereby adjusting the surface potential and increasing electrostatic repulsion. This enhances particle dispersion and improves flowability. However, when the NaCl dosage becomes excessive, according to DLVO theory, the excessively high ionic concentration sharply compresses the thickness of the electric double layer, shortening the effective range of electrostatic repulsion between particles. As a result, secondary flocculation of particles occurs, leading to an increase in paste viscosity [37,38].

#### 3.1.2. Setting Time

**Figure 5:**
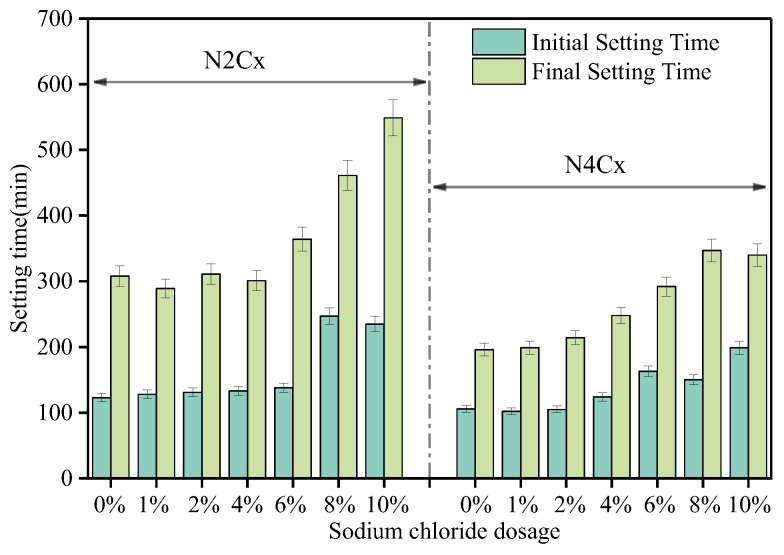
Effects of alkali equivalent (Na_2_O%) and NaCl dosage on the setting time of AAS.

Figure 5 shows the effect of alkali equivalent (Na_2_O%) and NaCl dosage on the setting time of the AAS. Under the low alkali equivalent condition (2%), the incorporation of 1 wt% NaCl can shorten the final setting time of the system. When the NaCl dosage is in the range of 1–4 wt%, there is no significant change in the initial and final setting times of the system. However, when the NaCl dosage exceeded 4 wt%, both the initial and final setting times of the paste were significantly prolonged. Furthermore, as the NaCl dosage increases, the setting times exhibit a more pronounced delay. When the NaCl dosage is 10 wt%, the initial setting time is extended from 123 min (for the baseline, N2) to 250 min, and the final setting time is extended from 308 min to 549 min.

Under the high alkaline equivalent condition (4%), 1 wt% NaCl also slightly accelerated setting, reducing both the initial and final setting times. At 2 wt% NaCl, the setting times were largely unaffected. However, when the NaCl dosage exceeds 4%, the setting time of the system gradually increases. For mix N4C10, the initial setting time is extended from 106 min to 199 min, and the final setting time is extended from 196 min to 340 min. This trend is consistent with the flowability test results, primarily because at this point, the ionic strength of the system is too high. The early-stage low-polymerized silicoaluminate species generated are difficult to further polymerize in the high-ion concentration environment, which hinders the formation of gel-like products, resulting in the macroscopic extension of the setting time. Comparing the alkaline-activated slag systems under low alkalinity (2%) and high alkalinity (4%), it can be observed that when the NaCl dosage exceeds 4 wt%, it shows a more significant retarding effect in both systems. This indicates that when the NaCl dosage is ≥4%, it has a significant setting delay effect under both low and high alkalinity conditions, and the regulation pattern of setting behavior tends to be consistent.

### 3.2. Compressive Strength

**Figure 6:**
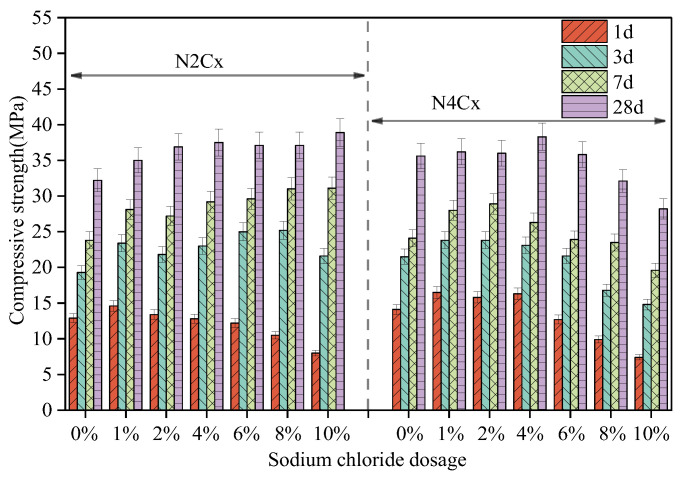
Effects of alkali equivalent and NaCl dosage on the compressive strength of AAS mortar.

Figure 6 shows the variation in compressive strength of alkaline-activated slag mortar with different alkalinity equivalents and NaCl dosages. Under low alkalinity equivalents (2%), the compressive strength of the mortar specimens with different NaCl dosages at 1-day curing age increases from 12.9 MPa (reference, N2) to 14.6 MPa (N2C1) and 13.4 MPa (N2C2). However, when the NaCl dosage exceeds 2 wt%, the 1-day compressive strength is lower than that of the NaCl-free group. This is consistent with the setting time trend, indicating that under low alkalinity conditions, the ionic effects of high NaCl concentration inhibit the early dissolution of slag and subsequent nucleation. At 3 days of curing, the strength of each NaCl dosage group (1–10 wt%) increased by 21%, 13%, 19%, 30%, 31%, and 12%, respectively. At 7 days, the strength increased by 18%, 14%, 23%, 24%, 30%, and 31%, respectively. At 28 days, the strength increased by 9%, 15%, 16%, 15%, 15%, and 21%, respectively. Under high alkalinity (4%) conditions, the mortar specimens with 1–4 wt% NaCl showed a 1-day strength increase from 14.1 MPa in the NaCl-free group to 16.5 MPa, 15.8 MPa, and 16.3 MPa, respectively. This is likely due to the fact that a moderate dosage of NaCl improved the flowability of the paste, thereby enhancing the molding quality of the specimens. When the NaCl dosage exceeds 4 wt%, the 1-day strength begins to decrease. At 3 days, the strength increased by 11%, 11%, and 7% for NaCl dosages of 1–4 wt%, respectively, while the strength was lower than that of the NaCl-free group when the NaCl dosage exceeded 4%. At 7 days, the strength increased by 16%, 20%, and 9% for NaCl dosages of 1–4 wt%, respectively, but it was still lower than that of the NaCl-free group when the NaCl dosage exceeded 4%. At 28 days, the strength increased by 2%, 1%, 8%, and 1% for NaCl dosages of 1–6 wt%, respectively, but when the NaCl dosage exceeded 6 wt%, the strength started to fall below that of the NaCl-free group.

In summary, for the low-alkali (2%) system, incorporating 1–10 wt% NaCl enhanced the compressive strength of the AAS mortar at 3, 7, and 28 days. However, at 1-day curing, when the NaCl dosage exceeds 1 wt%, the strength gradually decreases as the NaCl dosage increases. In the high-alkali (4%) system, the incorporation of 1–4 wt% NaCl improves the compressive strength of the AAS mortar at all ages. However, when the NaCl dosage exceeds 4 wt%, the strength at all curing ages is lower than that of the reference group. The above strength variation trend reflects that the effect of NaCl on the performance of AAS is closely related to the alkalinity. This variation may be associated with the hydration process, the composition of hydration products, and the evolution of the microstructure. The specific reasons will be elucidated in the following sections, in conjunction with the subsequent experiments.

### 3.3. Phase Composition

#### 3.3.1. XRD

**Figure 7:**
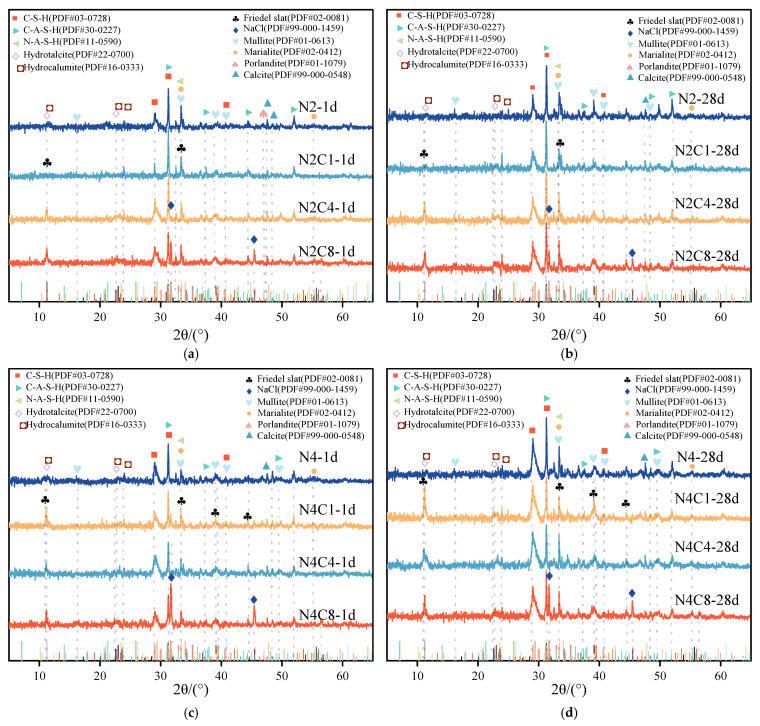
XRD patterns of AAS at 1 d and 28 d with varying alkali equivalents and NaCl dosages. (**a**) N2Cx-1d; (**b**) N2Cx-28d; (**c**) N4Cx-1d; (**d**) N4Cx-28d.

Figure 7 shows the XRD patterns of hardened pastes at 1 d and 28 d. All samples exhibit a broad diffraction hump centered at approximately 30° (2θ), characteristic of low-crystallinity C-(A)-S-H gel [39]. This gel phase provides the main strength and contributes to microstructural densification [40]. The hump intensity increases from 1 d to 28 d, indicating continuous hydration and gel formation. Compared to the N2 group, the N4 group shows higher hump intensity, confirming that higher alkalinity promotes slag dissolution and accelerates gel formation. Crystalline phases, including calcite (PDF#99-000-0548), mullite (PDF#01-0613), and marialite (PDF#02-0412), are also detected. Calcite likely originates from carbonation during paste hardening [41].

In all NaCl-containing samples, layered double hydroxides such as Friedel’s salt (PDF#02-0081), hydrotalcite (PDF#22-0700), and hydrocalumite (PDF#16-0333) are observed, indicating that Al^3+^ and Ca^2+^ react with Cl^−^ to form chloride-bearing AFm phases [42]. These phases play a key role in chloride immobilization and microstructural regulation.

At 1 d, the effect of NaCl on early hydration depends strongly on alkalinity. In the low-alkali system (2% Na_2_O), the C-(A)-S-H diffraction peak increases with 1–4% NaCl, indicating that moderate NaCl promotes early hydration. In contrast, in the high-alkali system (4% Na_2_O), the C-(A)-S-H peak intensity decreases with increasing NaCl dosage, suggesting competitive inhibition. When NaCl dosage exceeds 8%, distinct NaCl diffraction peaks appear. These NaCl crystals may partially precipitate during sample preparation and do not fully represent the in-situ hydration state. However, their presence correlates with strength reduction, indicating that excessive NaCl adversely affects material performance [43].

At 28 d, the C-(A)-S-H and N-A-S-H gel peaks increase in all samples compared to 1 d. Friedel’s salt intensity increases with NaCl dosage up to 4% but decreases at 8%, possibly due to NaCl recrystallization. In the low-alkali system (2% Na_2_O), NaCl promotes slag dissolution and Friedel’s salt formation. The plate-like Friedel’s salt crystals act as micro-fillers, bridging the gel and optimizing pore structure [44]. This explains the continuous strength increase with NaCl dosage at later ages in the N2 system.

In the high-alkali system (4% Na_2_O), Friedel’s salt is also detected, but NaCl diffraction peaks appear simultaneously, indicating that excess Cl^−^ exists as NaCl crystals. Under high alkalinity, NaCl precipitation may cause microstructural damage and inhibit slag dissolution. Excessive Cl^−^ may also promote transformation of C-S-H into less stable phases, such as Friedel’s salt, hindering normal gel formation [45] and leading to significant strength reduction.

#### 3.3.2. FTIR

**Figure 8:**
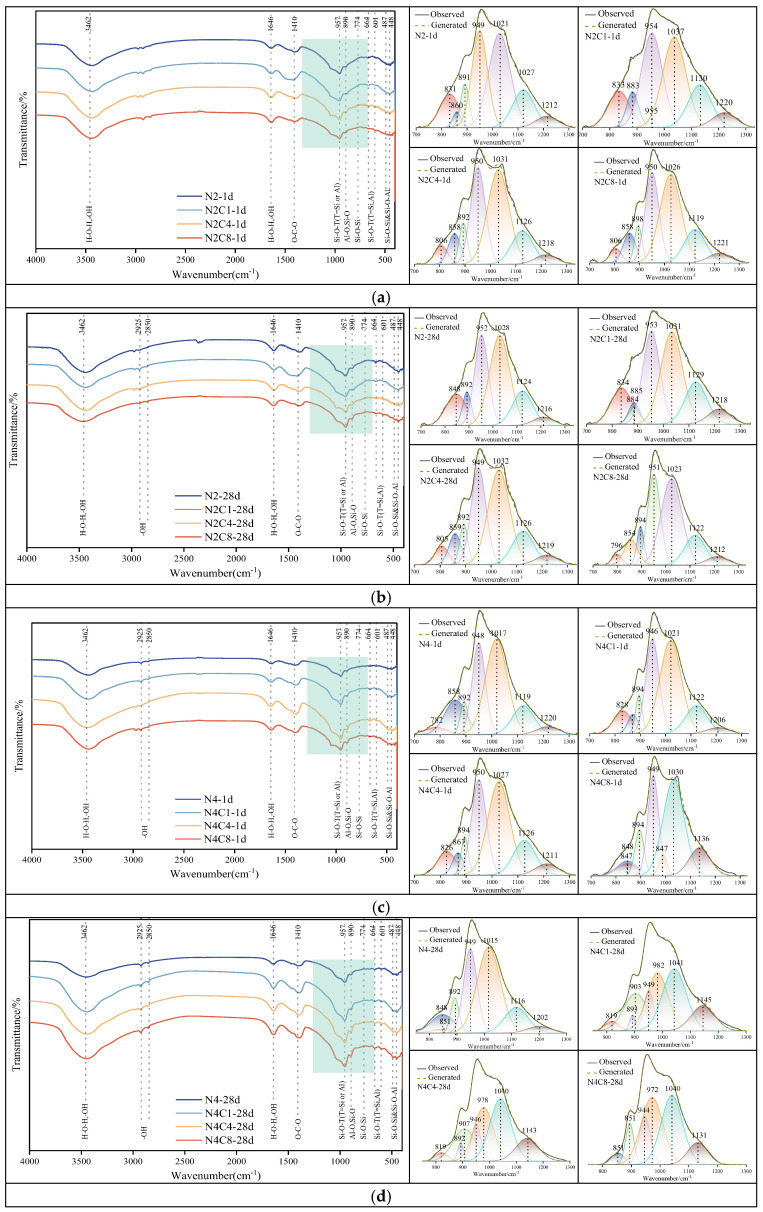
FTIR spectra of AAS at 1 d and 28 d with different alkali equivalents and NaCl dosages. (**a**) N2Cx-1d; (**b**) N2Cx-28d; (**c**) N4Cx-1d; (**d**) N4Cx-28d.

Figure 8 shows the FTIR spectra of hardened paste at 1-day and 28-day curing ages with different alkali equivalents and NaCl dosages, as well as the deconvolution fitting results of the Si-O-T stretching vibration peak in the 800–1200 cm^−1^ range. In the spectra, the absorption bands in the 850–1100 cm^−1^ range are related to the antisymmetric stretching vibration of the Si-O-T (T = Si or Al) bonds in the C-A-S-H gel [46]. In the low-alkali (2%) system at 1 day, the sample with 1 wt% NaCl shows an increased intensity in this region of the spectrum, indicating that a low dosage of NaCl promotes the early dissolution of slag and the formation of gel phases. At 28 days of curing, the intensity of the Si-O-T antisymmetric stretching vibration peak decreases, suggesting that the incorporation of NaCl inhibits the polymerization of the gel phase. In the high-alkali (4%) system, at all curing ages, the addition of NaCl increases the intensity of the absorption band in this region, indicating that in the high-alkaline environment, the incorporation of NaCl alters the reaction pathway and promotes the formation of silicate structures with higher polymerization [47]. The band near 1420–1440 cm^−1^ is characteristic of the O-C-O asymmetric stretching vibration in carbonate species, arising from the carbonation of alkali oxides [46]. Its presence confirms that some degree of atmospheric carbonation occurred, consistent with the XRD findings.

The absorption bands in the 1600–1700 cm^−1^ and 3000–3500 cm^−1^ ranges correspond to the vibrations of crystallization water and physically adsorbed water, respectively [46]. These water molecules exist in the channels or cavities of the geopolymer, and the intensity of their absorption peaks increases as the degree of hydration in the system grows. This also reflects the increasing quantity of hydration products as the hydration age progresses [48]. In the low-alkali (2%) system, the incorporation of NaCl has little effect on the intensity of the above absorption bands at 1 day of curing. However, at 28 days of curing, the intensity of these absorption bands becomes more pronounced with the addition of 1–4 wt% NaCl. In the high-alkali system, these water bands are significantly more intense. This could be linked to a higher degree of gel formation and a distinct gel structure under high alkalinity. Notably, the O-H stretching region (3000–3500 cm^−1^) is sensitive to the hydrogen bonding environment within the C-A-S-H gel [46]. The enhanced intensity in this region with NaCl addition might, therefore, indicate alterations in the gel’s bonding characteristics, which could correlate with the observed mechanical performance.

**Figure 9:**
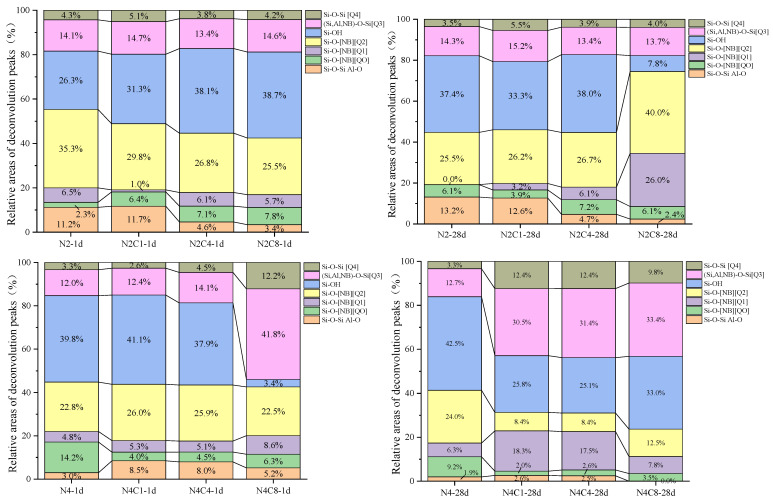
Relative distribution of Q^n^ units from deconvoluted FTIR spectra of AAS at 1 d and 28 d.

To further investigate the effect of NaCl incorporation on the structural evolution of the silicate network, the key region between 800 and 1200 cm^−1^ was subjected to deconvolution, and the fitting results are shown in Figure 9. The purpose of this method is to deconvolve the severely overlapping absorption bands, thereby semi-quantitatively analyzing the distribution of Q^n^ units that represent different degrees of polymerization, providing direct structural parameters for understanding the regulatory mechanism of NaCl.

Figure 9 presents the deconvolution results of the main Si-O-T (T = Si or Al) stretching band (800–1300 cm^−1^) for pastes with different alkali equivalents and NaCl dosages, quantifying the distribution of silicate Q^n^ units. The deconvoluted sub-bands were assigned based on the established literature.

795–814 cm^−1^: Symmetric Si-O-Si stretching in residual quartz/crystalline phases and Al-O vibrations [49].

850–880 cm^−1^: Si-O vibrations of non-bridging oxygen (NBO, Si-O-) in monomeric/dimeric (Q0,Q1) silicate species [50].

900–930 cm^−1^: Characteristic of dimeric/polymeric (Q1) silicate chains [50].

950–990 cm^−1^: Stretching of Si-O in chain-like (Q2) structures and Si-OH groups [50,51].

1048–1100 cm^−1^: Asymmetric stretching of (Si, Al)-O-Si in sheet-like (Q3) structures [50].

1162–1200 cm^−1^: Asymmetric Si-O-Si stretching in three-dimensional network (Q4) structures [50].

In the low-alkali (2%) system at 1 day, low NaCl dosage promotes structural reorganization, while high NaCl dosage inhibits polymerization. From the figure, it can be observed that when 1% NaCl is incorporated, the content of Q^2^ units decreases from 35.34% to 29.79%, and the ratio of Q^0^ + Q^1^ increases, indicating that the incorporation of 1 wt% NaCl accelerates the early hydration rate, providing a foundation for the rapid formation of mineral gels [52]. At the same time, the ratio of Q^3^ to Q^4^ shows a slight increase, reflecting that the incorporation of 1 wt% NaCl promotes the structural transformation towards a higher degree of polymerization [52]. When 4% NaCl is incorporated, Q^2^ further decreases to 26.75%, while the Si-OH content increases to 38.14%, indicating an increase in the gel’s intermediate phases and an accelerated polymerization reaction [53]. When 8% NaCl is incorporated, Q^2^ continues to decrease to 25.53%, while the Si-OH content hardly decreases, indicating that at high NaCl dosages, monomer condensation is hindered, and the structural development is incomplete. At 28 days, in the N2C8 group, the Si-OH content decreased to 7.79%, accompanied by an increase in the ratio of Q^0^ + Q^1^ to 32.14%. This indicates that a high NaCl dosage inhibits the later-stage polymerization of the gel phase. In the high-alkali (4%) system, at 1 day of age, Q^2^ in the N4 group is 22.8%, and it slightly increases to about 26% when NaCl dosages are 1–4 wt%. However, the ratio of Q^0^ + Q^1^ is relatively high, indicating that NaCl incorporation inhibits the formation of the gel phase. At 8 wt% NaCl, an increase in Q^3^ alongside a decrease in Q^2^ suggests a shift towards a more cross-linked but possibly shorter-chained or disordered network, which correlates with the observed strength reduction. This observation aligns with findings by Yuan et al. [54], who reported that a high early degree of cross-linking can slow down subsequent reaction kinetics, delaying strength development.

At 28 days of curing, the ratio of Q^3^ + Q^4^ is relatively high (43.25%), but the Q^2^ content is very low (12.49%), and the Si-OH content is relatively high. This Q^n^ distribution is indicative of a disordered, highly cross-linked but poorly connected gel network, which explains the significant strength decrease at high NaCl dosages.

#### 3.3.3. TG-DTG

**Figure 10:**
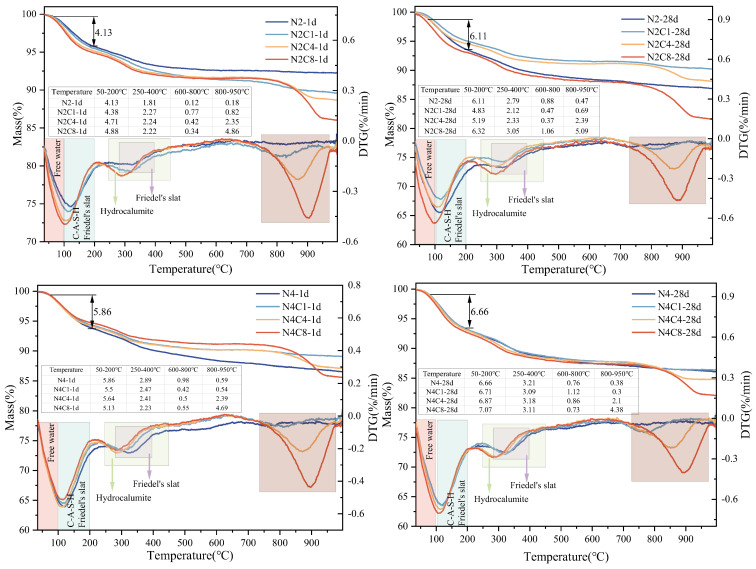
TG-DTG curves of AAS at 1 d and 28 d under different alkali equivalents and NaCl dosages.

To further analyze the effect of NaCl dosage at different curing ages on the phase composition of AAS, thermogravimetric tests were conducted, and the results are shown in Figure 10. The DTG curve shows that the thermal weight loss of the system mainly includes: free water, physically adsorbed water, structural water in the C-(A)-S-H gel, and surface and interlayer water removal from Friedel’s salt in the range of 50–200 °C [42,55]; the mass loss between 250 and 400 °C is mainly related to the dehydroxylation of hydrocalumite and Friedel’s salt [42,56], the mass loss between 600 and 800 °C is primarily due to the decomposition of CaCO_3_ [55,57], and the mass loss between 800 and 950 °C is primarily associated with the further decomposition of Friedel’s salt.

Under low-alkali dosage conditions (2%), the incorporation of NaCl into the alkali-activated slag system can significantly promote the early hydration of cementitious materials. It was observed that at a curing age of 1 day, the endothermic peak near 100 °C increased markedly with increasing NaCl content, indicating that NaCl addition accelerated early hydration and enhanced the formation of C-A-S-H gel and Friedel’s salt in the system [58,59]. This is consistent with the results of workability and strength tests, suggesting that the improvement in early hydration contributes to better material performance. At 28 days, the N2C8 group exhibited a significantly higher peak near 100 °C than the reference N2 group; combined with the XRD results, this confirms the stable presence of Friedel’s salt in the system. The dehydration and dehydroxylation processes in the 50–200 °C and 250–400 °C ranges of the TG–DTG curves are related to interlayer water and structural water in the AFm-type layered structure. With increasing NaCl content, the total weight loss of the system showed an upward trend, indicating that chloride ions were chemically incorporated into the layered structure rather than merely physically filling it, thereby increasing the chemically bound water in the system [60]. Ansair et al. studied the influence of calcium sulfoaluminate cement on the performance of cementitious materials and pointed out that the increase in the total mass loss rate in TG analysis is related to the rise in the total amount of hydration products. The changes in the peak area of characteristic peaks can be used to estimate the variation in the content of specific phases. In this study, the increase in total weight loss and the enhancement of Friedel’s salt-related peak intensity both support the conclusion that chloride ions participated in chemical binding and formed stable hydration products [61]. This aligns with the increasing trend of 28-day compressive strength and further confirms that under low-alkali dosage, NaCl effectively promotes the later-stage hydration of the system, thereby enhancing the mechanical properties of the material.

In the high-alkali composite system, the overall weight loss was higher than that of the low-alkali N2 group, indicating a greater degree of hydration under high-alkali conditions [62]. However, unlike under low-alkali conditions, with increasing NaCl content, the secondary decomposition peak of Friedel’s salt in the 800–950 °C range increased significantly, while the width and intensity of the peak near 100 °C showed no noticeable change. This indicates that although high NaCl dosages under high-alkali conditions promote the formation of Friedel’s salt, their effect on early hydration is less pronounced than under low-alkali conditions [63]. Combined with the strength test results, it can be inferred that under high-alkali conditions, excessive NaCl addition may lead to increased concentrations of free Cl^−^, inhibiting the polymerization of C-A-S-H gel. At the same time, Na^+^ may competitively incorporate into AFm phases or precipitate as NaCl crystals, thereby weakening the integrity of the gel network.

Overall, as the NaCl content in the system increases, the decomposition peak area of Friedel’s salt enlarges, confirming that chloride ions in the system are not merely physically filling voids but participate in chemical reactions to form Friedel’s salt and hydrotalcite-like phases, thereby increasing the chemically bound water in the system. Chloride ions themselves can also be effectively immobilized within the LDH structures. LDHs efficiently bind chloride ions through multiple mechanisms, including physical adsorption, ion exchange, and interlayer fixation, thereby reducing the concentration of free chloride ions in the system [64,65]. At a curing age of 28 days, the decomposition peaks of hydrocalumite in the N2C8 and N4C8 groups increased, which may be attributed to the incorporation of NaCl altering the existing forms of aluminum phases in the system. The occurrence of this phenomenon is similar to the finding that calcium sulfoaluminate cement particles alter the distribution of hydration products and the phase composition of the interfacial transition zone (ITZ) by optimizing particle gradation and the filling effect [61]. In this study, the incorporation of NaCl similarly altered the occurrence state of the aluminum phase in the system, promoting the formation of layered double hydroxides, thereby affecting the microstructure and phase composition of the system.

### 3.4. Microstructure

**Figure 11:**
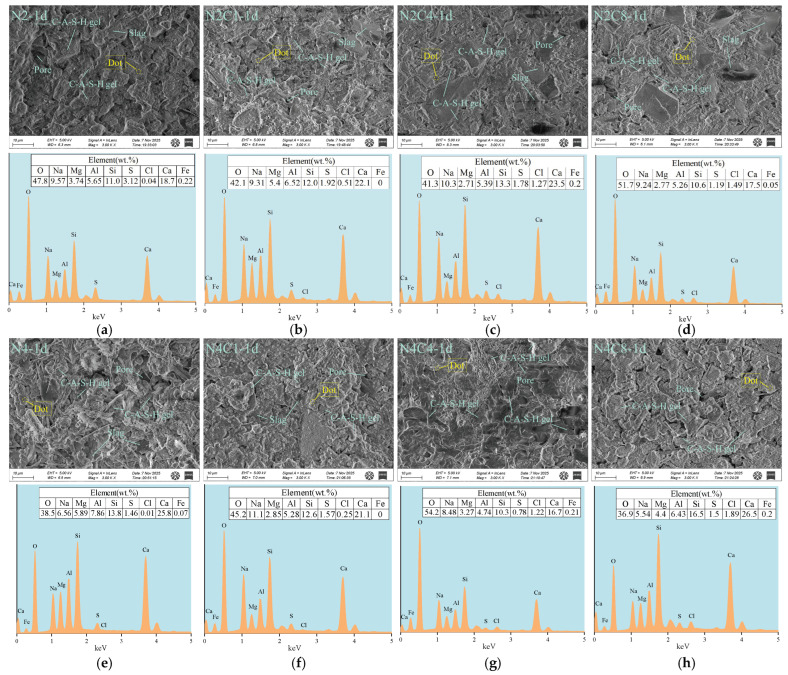
SEM-EDS analysis of AAS at 1 d with different alkali equivalents and NaCl dosages. (**a**) N2-1d; (**b**) N2C1-1d; (**c**) N2C4-1d; (**d**) N2C8-1d; (**e**) N4-1d; (**f**) N4C1-1d; (**g**) N4C4-1d; (**h**) N4C8-1d.

Figure 11 shows the SEM-EDS images of hardened paste at 1-day age with different alkali equivalents and NaCl dosages. The SEM-EDS analysis results at 1 day indicate that different alkali equivalents and NaCl dosages significantly regulate the composition and microstructural evolution of the early hydration products in the alkali-activated slag system.

In the low-alkali equivalent (2%) system, the microstructure exhibits a regular change with the increase in NaCl dosage. In the control group without NaCl (Figure 11a), a considerable number of unhydrated slag particles are dispersed in the paste. The C-(A)-S-H gel network is poorly developed, and the cement paste structure is loose, indicating a low degree of early hydration. When 1 wt% NaCl is added (Figure 11b), the unhydrated particles decrease, and the C-(A)-S-H gel forms a continuous and dense network structure, indicating that an appropriate amount of NaCl effectively promotes slag dissolution and the hydration process. However, when the NaCl dosage is too high, as shown in (Figure 11c,d), the number of unhydrated particles in the paste increases compared to the control group, and the development of the gel structure is significantly hindered, indicating that excessive Cl^−^ has an inhibitory effect on early hydration. EDS analysis further reveals the binding behavior of chloride ions. At a NaCl dosage of 8 wt%, the Cl/Al ratio in the gel phase increases to 0.283, indicating that more chloride ions are being incorporated into the gel matrix within the reaction zone.

In the high-alkali equivalent (4%) system, the addition of NaCl shows a more pronounced inhibitory effect on early hydration. As the NaCl dosage increases, the gel structure gradually becomes looser. The EDS results show that under high-alkali equivalent (4%) conditions, the Cl/Al ratio varies significantly (0.001–0.294) as the NaCl dosage increases and is generally higher than in the low-alkali group. This indicates that under high alkalinity conditions, the interaction between chloride ions (Cl^−^) and aluminum ions (Al^3+^) is strengthened, which significantly affects the formation and structural development of C-(A)-S-H gel in the cement-based material. This effect is primarily attributed to the chloride–aluminum binding process, which competitively consumes the key aluminum source involved in the gelation reaction [66], resulting in a reduction in the quantity of raw materials available for gel formation. This is consistent with the enhancement of Friedel’s salt diffraction peak in XRD and the changes in related bond positions in FTIR, which together confirm the physical adsorption and chemical entrapment mechanisms of chloride ions in the gel.

**Figure 12:**
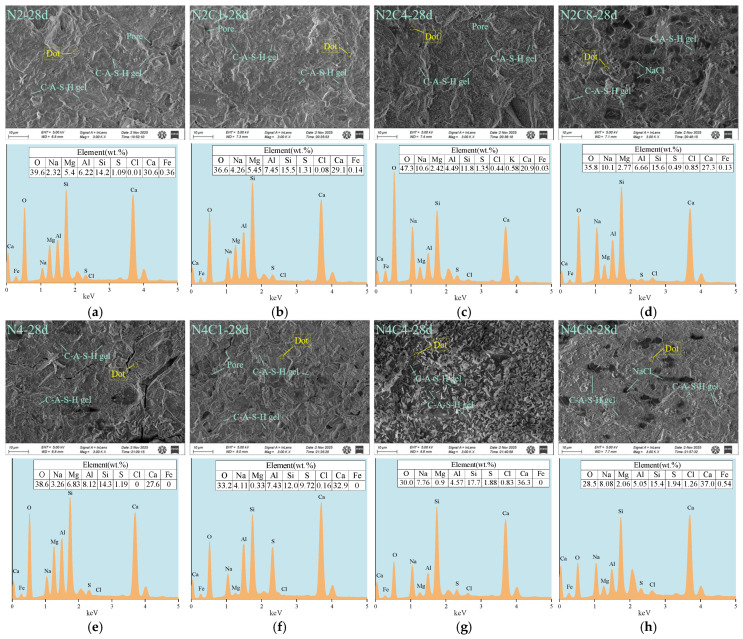
SEM-EDS analysis of AAS at 28 d with different alkali equivalents and NaCl dosages. (**a**) N2-28d; (**b**) N2C1-28d; (**c**) N2C4-28d; (**d**) N2C8-28d; (**e**) N4-28d; (**f**) N4C1-28d; (**g**) N4C4-28d; (**h**) N4C8-28d.

Figure 12 shows the SEM-EDS images of hardened paste at 28 days of age under different alkali equivalents and NaCl dosages. As seen in the figure, the different alkali equivalents and NaCl dosages continue to exert a regulating effect on the long-term microstructural evolution and product composition of the alkali-activated slag system.

In the low-alkali (2%) system, compared to the 1-day age, the composition of the cured products at 28 days undergoes significant changes. EDS analysis shows that the Cl/Al ratio continuously increases with the NaCl dosage (0.002–0.128), indicating that chloride ions are continuously fixed in the C-A-S-H gel throughout the curing period. From Figure 12a–c, it can be observed that the C-(A)-S-H gel structure is uniform and continuous in the samples without NaCl. However, after the addition of NaCl, plate-like Friedel’s salt crystals appear distinctly in the samples. These crystalline phases fill the pores between the gel, enhancing the compactness of the matrix. This micro-filling effect explains the significant increase in compressive strength in the N2C10 group.

In the high-alkali (4%) system, the inhibitory effect of NaCl on the later-term hydration is more pronounced. The Cl/Al ratio increases from 0.000 to 0.250, indicating that as the NaCl dosage increases, more chloride ions are fixed within the generated C-(A)-S-H gel. The SEM images in Figure 12f–h show that even a low amount of NaCl induces significant precipitation of Friedel’s salt. As the NaCl dosage increases, both the crystal size and quantity increase. In the N4S8 group, a large number of cubic crystals were observed (Figure 12h), and EDS analysis confirmed that their main elements are Na and Cl, indicating that they are NaCl crystals. This phenomenon may result from the precipitation of NaCl crystals during the drying process, which also reflects that high concentrations of Na^+^ and Cl^−^ were retained in the pore solution. These unimmobilized ions crystallized during drying, indirectly demonstrating that the chloride fixation efficiency is low in high-alkali slag systems. The presence of these free ions may affect the hydration equilibrium by increasing the ionic strength of the pore solution or generating crystallization stress under drying conditions. Moreover, the formed NaCl crystals act almost like pore defects in terms of mechanical properties, and their presence leads to a decrease in the overall compactness and mechanical strength of the hardened paste.

The above analysis indicates that the inhibitory effect of NaCl on the development of C-A-S-H gel persists during long-term curing, and the continuous formation of Friedel’s salt significantly competes with the structural development of the gel phase. The Cl/Al ratio can serve as a key indicator for assessing the degree of chloride ion fixation [67]. An increase in this ratio reflects the continuous fixation of chloride ions in C-A-S-H and Friedel’s salt, but it is also accompanied by a weakening effect on the dense gel network. This result provides important microstructural evidence for understanding the long-term performance effects of NaCl in alkali-activated systems.

### 3.5. Discussion

**Figure 13:**
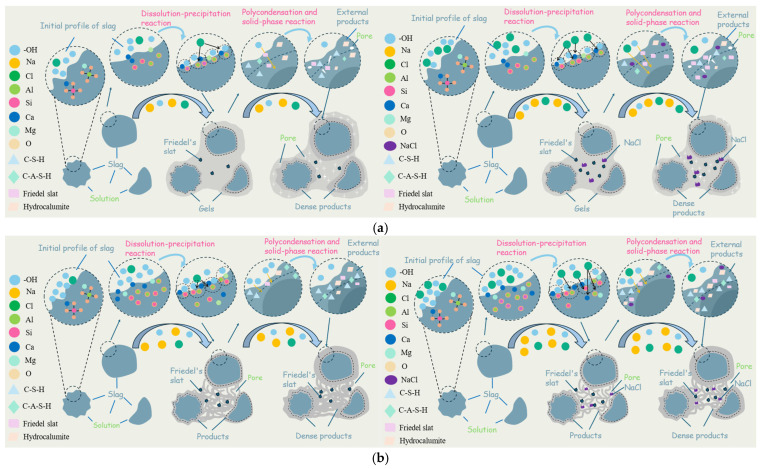
Schematic diagram of the influence of alkali equivalent and NaCl content on the slag reaction process. (**a**) Schematic diagram illustrating the hydration mechanism of AAS under the low-alkali equivalent with different NaCl dosages. (**b**) Schematic diagram illustrating the hydration mechanism of AAS under the high-alkali equivalent with different NaCl dosages.

In the NaCl-NaOH-activated slag system, the aluminosilicate glassy phase depolymerizes under alkaline conditions, releasing SiO_4_^4−^, AlO_4_^5−^, and Ca^2+^ ions that undergo polycondensation to form C-A-S-H gel. The coupled effects of alkali equivalent and NaCl dosage significantly influence ion dissolution dynamics, product competition, and microstructural evolution. These mechanistic differences are schematically illustrated in Figure 13, which compares the reaction pathways under low and high alkalinity with varying NaCl dosages.

As shown in Figure 13a, under low alkalinity (2% Na_2_O), slag depolymerization is relatively slow. The introduction of an appropriate amount of NaCl increases ionic strength, compressing the electrical double layer on slag particle surfaces [58]. According to DLVO theory, this compression maintains sufficient electrostatic repulsion while reducing effective collision distance, enhancing particle dispersion within the paste [37]. This mechanism promotes early hydration, as confirmed by FTIR deconvolution showing increased Q^0^ + Q^1^ units at 1 d, macroscopically manifesting as improved flowability and early strength. At later stages, moderate NaCl induces Friedel’s salt formation, which coexists synergistically with C-A-S-H gel. As illustrated in the schematic, plate-like Friedel’s salt crystals fill pores, acting as micro-fillers that refine microstructure, explaining the continuous strength increase in the N2 series with NaCl dosage at later ages. However, when NaCl exceeds 4 wt%, excessive AlO_4_^5−^ preferentially reacts with Cl^−^ to form Friedel’s salt, competitively consuming aluminum sources required for C-A-S-H formation, leading to reduced early strength.

Figure 13b depicts the reaction pathway under high alkalinity (4% Na_2_O). High OH^-^ concentration rapidly dissolves slag particles, creating a high ion supersaturation zone where SiO_4_^4-^ rapidly polymerizes with Ca^2+^ and Al^3+^ to form C-A-S-H gel. However, when high Cl^−^ concentrations are introduced, competition between Cl^−^ and AlO_4_^5+^ significantly affects C-A-S-H formation [66]. The schematic illustrates how excessive Cl^−^ preferentially binds with Al^3+^ to form Friedel’s salt and other AFm phases, disrupting the primary gel network. Combined with NaCl crystallization, this leads to microstructural damage, with strength decreasing as NaCl dosage increases. Notably, the significant retarding effect caused by high NaCl dosages (>4%) may have adverse implications for practical engineering applications.

From a durability perspective, NaCl incorporation may exert dual effects on chloride resistance and carbonation behavior. Microscopic evidence confirms Friedel’s salt and Cl^−^ containing AFm phases formation, with Cl/Al ratios increasing proportionally with NaCl dosage, indicating partial chloride immobilization in hydration products. This chemical binding theoretically reduces free Cl^−^ concentration in pore solution, enhancing resistance to external chloride ingress. Under low alkalinity, moderate NaCl promotes C-A-S-H gel densification and improves later-age strength, suggesting synergistic effects of microstructure refinement and chloride binding that may benefit long-term durability. Conversely, under high alkalinity with excessive NaCl, disturbed hydration kinetics and microstructural damage imply potential adverse effects on durability. Additionally, chloride ions may alter pore solution chemistry and buffering capacity, potentially influencing carbonation resistance. Industrial brine contains multiple coexisting ions whose interaction mechanisms may differ from pure NaCl systems; therefore, the proposed alkali-dependent mechanisms require validation under realistic mixed-salt conditions through combined durability testing and thermodynamic analysis.

From an environmental standpoint, AAS systems offer inherent advantages by eliminating clinker calcination and utilizing industrial by-products. However, conventional activators (NaOH, water glass) are energy-intensive and contribute significantly to the carbon footprint. This study demonstrates that under low-alkali conditions (2% Na_2_O), partial replacement of NaOH with NaCl (up to 4%) maintains or enhances later-age mechanical performance while promoting slag reaction and gel densification. This strategy theoretically reduces activator-related CO_2_ emissions. Furthermore, the system shows promise for valorizing industrial saline wastewater (primarily NaCl), as evidenced by chemical incorporation of Cl^−^ into Friedel’s salt and hydrotalcite-like phases, reducing the risk of free chloride release. Nevertheless, a comprehensive life cycle assessment was not conducted, and net environmental benefits require systematic quantification in future studies.

Several methodological limitations should be acknowledged. Chloride binding was assessed semi-quantitatively using EDS-derived Cl/Al ratios; while providing useful insights into relative chloride fixation, more precise quantification methods (e.g., selective dissolution, leaching tests, or total chloride analysis) are needed to confirm absolute binding capacity and long-term stability. Mercury intrusion porosimetry (MIP), isothermal calorimetry, and thermodynamic simulations were not performed; therefore, discussions on pore structure evolution and hydration kinetics rely primarily on SEM observations and literature comparisons. XRD analysis was not quantified by Rietveld refinement, FTIR deconvolution is inherently semi-quantitative, and TG-DTG reflects total mass loss without phase separation. Consequently, the results should be interpreted as relative trends rather than absolute quantitative conclusions. Although FTIR deconvolution followed a standardized procedure and SEM-EDS data were averaged over multiple regions, the conclusions remain qualitative or semi-quantitative.

Future research should integrate MIP, isothermal calorimetry, thermodynamic modeling (e.g., PHREEQC), and Rietveld quantitative XRD to obtain a more comprehensive understanding of reaction mechanisms and phase evolution in NaCl-NaOH-activated slag systems. Long-term durability studies (chloride penetration, carbonation, steel corrosion) under realistic exposure conditions are essential to validate practical applicability. Additionally, the proposed mechanisms should be verified using real industrial saline wastewater containing multiple coexisting ions to assess engineering feasibility.

## 4. Conclusions

This study systematically investigated the effects of alkali equivalent (Na_2_O%) and NaCl dosage on the workability, mechanical properties, and microstructure of alkali-activated slag cement. The main conclusions are as follows:

1. In the low-alkali (2%) system, incorporating an appropriate amount of NaCl (≤4 wt%) enhances ionic strength, promoting slag particle dispersion and dissolution. The introduced Cl^−^ ions participate in forming Friedel’s salt, which refines the matrix porosity. These combined effects improve early-age workability and strength. However, beyond 4 wt%, the beneficial effects diminish with further increases in NaCl dosage.
2. In the high-alkali (4%) system, NaCl addition leads to preferential binding of Cl^−^ with early-released Al^3+^ and Ca^2+^. This competitively inhibits the formation of the primary C-(A)-S-H gel, resulting in a more porous microstructure. At excessively high dosages, NaCl recrystallization occurs. The resultant NaCl crystals act as physical defects, degrading matrix continuity and leading to strength deterioration.
3. This work clarifies the dual role of NaCl in NaOH-activated slag systems: it can function as an auxiliary activator at optimal dosages but becomes detrimental in excess. Its beneficial effect is particularly pronounced in low-alkali systems, where it significantly enhances performance by promoting hydration and refining microstructure.

## Figures and Tables

**Table 1:** Research on neutral salt–alkali-activated cementitious systems.

Precursor Type	Excitant	Excitant Dosage	Hydration Products	References
Slag + Fly Ash	CaO + MgO	10%	C-(A)-S-H/Aft	Gu K (2014) [10]
	Ca(OH)_2_ + CaSO_4_·2H_2_O /NaOH/Na_2_CO_3_/Na_2_SO_4_	6.25%	C-S-H/AFm/AFt/Ht	Jeong Y (2016) [11]
	Na_2_SO_4_	5%/10%/25%	Aft/C-A-S-H/Ht	Mobasher N (2016) [12]
	Ca(OH)_2_ + Na_2_CO_3_	0–7.5%	C-S-H/Ht	Wang J (2018) [13]
	MgO + NaOH	--	C-A-S-H/Ht	Burciaga-Díaz O (2018) [14]
	Na_2_CO_3_/Na_2_SO_4_	0–5%	Calcite/AFt	Tan H (2019) [15]
	Ca(OH)_2_ + NaSO_4_	0–4%	C-S-H/AFt/CH	Wu M (2019) [16]
	Na_2_SO_4_ + CaO	0–8% + 20%	C-S-H/Aft	Zhao Y (2020) [17]
	NaOH/NaS	14.6%/36.2%	C-S-H/AFt	Biricik H (2021) [18]
	MgO	--	C-S-H/Ht/AFm	Zhou G (2025) [19]
	Alkali slag + Carbide slag	--	AFt/AFm/Ht/C-S-H	Zhang H (2025) [20]
Slag	CaCl_2_ + Carbide slag NaCl + Carbide slag	0–7%	C-S-H/Ht/Calcite	Li M (2023) [21]
	NaOH + Na_2_CO_3_ NaOH + Na_2_O·1.5SiO_2_	--	C-A-S-H/Calcite/Ht	Dai X (2023) [22]
	Quicklime	5–13%	C-S-H/CH/Ht	Chilmon K [23]
	Na_2_SO_4_ + Na_2_O·1.5SiO_2_ + NaOH	0–6%	C-A-S-H/Aft/Ht/Calcite/Gypsum	Yang C (2026) [24]

**Table 2:** Chemical properties of GBFS.

Oxide	SiO_2_	MgO	Al_2_O_3_	Fe_2_O_3_	SO_3_	K_2_O	Na_2_O	CaO	Fe_2_O_3_	LOI
wt%	33.64	12.19	16.93	0.40	2.46	0.35	0.78	31.84	0.40	0.09

**Table 3:** Mixture design of alkali-activated slag mixtures.

No.	Na_2_O (wt%)	NaCl (wt%)	[Cl^−^] (mol/L)	[Na^+^] (mol/L)	GBFS	w/b
N2	2	-	0	1.70	1	0.38
N2C1		1	0.45	2.15		
N2C2		2	0.90	2.60		
N2C4		4	1.80	3.50		
N2C6		6	2.70	4.40		
N2C8		8	3.60	5.30		
N2C10		10	4.50	6.20		
N4	4	-	0	3.40		
N4C1		1	0.45	3.85		
N4C2		2	0.90	4.30		
N4C4		4	1.80	5.20		
N4C6		6	2.70	6.10		
N4C8		8	3.60	7.00		
N4C10		10	4.50	7.90		

Note: N2 and N4 represent the reference groups with alkali equivalents of 2% and 4% Na_2_O, respectively. C1, C2, C4, C6, C8, and C10 denote NaCl dosages of 1%, 2%, 4%, 6%, 8%, and 10% by mass of slag, respectively.

## Data Availability

The original contributions presented in this study are included in the article. Further inquiries can be directed to the corresponding author.
